# Genetic heterogeneity and diversity of North American golden retrievers using a low density STR marker panel

**DOI:** 10.1371/journal.pone.0212171

**Published:** 2019-02-27

**Authors:** Eric S. Ontiveros, Shayne Hughes, Maria Cecilia T. Penedo, Robert A. Grahn, Joshua A. Stern

**Affiliations:** 1 Department of Medicine and Epidemiology, School of Veterinary Medicine, University of California Davis, Davis, California, United States of America; 2 Veterinary Genetics Laboratory, University of California Davis, Davis, California, United States of America; National Cheng Kung University, TAIWAN

## Abstract

Thirty-three autosomal short tandem repeat (STR) markers were used to evaluate genetic heterogeneity and diversity in 525 golden retrievers (GRs). This breed was selected because of its popularity and artificial selection for conformation vs. performance phenotypes. Seven additional STRs were used to evaluate the highly polymorphic dog leukocyte antigen (DLA) class I and class II regions. From 3 to 13 alleles were found at each of the 33 loci (mean 7) and the average effective alleles (Ne) was 3.34. The observed heterozygosity was 0.65 and the expected heterozygosity was 0.68. The resulting fixation index was 0.035 indicating that the population was randomly breeding. We found that modern GRs retain 46% of genomic diversity present in all canids and 21/175 (12%) and 20/90 (22%) of the known DLA class I and class II haplotypes, respectively. Selection for performance or conformation led to a narrowing of genomic and DLA diversity with conformation having a greater effect than performance. A comparison was made between coefficient of inbreeding (COI) determined from 10 or 12 generation pedigrees and DNA based internal relatedness values. A weak but significant correlation was observed between IR score and 10 or 12 generation COI (r = 0.38, p<0.0001 and r = 0.40, p<0.0001, respectively). IR values were higher in conformation than performance lines but only significant at p = 0.17. This was supported by 10 and 12 generation COI values that were significantly (p<0.0001) higher in conformation than performance lines. We demonstrate herein that a low density of STR markers can be utilized to study the genetic makeup of GRs.

## Introduction

Purebred dogs originate from a highly select group of founders that are pre-defined by a homogenous standard. Once a breed is registered, further gene flow is prohibited thus ensuring the purity and maintenance of the breed. Therefore, to continue to achieve a desired phenotype, many purebred dog breeders utilize line-breeding and may overuse popular sires [1]. Although the desire is to maintain a homogenous phenotype, a dog breed can often vary over time in their appearance within the allowance of the breed standard. Furthermore, phenotypic variation can result due to geographic isolation or intentionally by positive human-directed selection [2]. Artificial selection pressure on dog breeds can also involve breeding for a specific conformation trait or a performance trait. This may result in periods of inbreeding that can have an effect on the genetic heterogeneity and/or diversity that existed in the founder population. Dog breeders are therefore tasked to avoid inbreeding due to its possible effect on genetic diversity that can result in an increased incidence of inherited diseases [3, 4].

To help maintain the genetic diversity of purebred dogs, breeders have relied on pedigree-derived coefficient of inbreeding (COI) percentages calculated over a few recent generations. One general purpose of utilizing a COI percentage is to produce healthier offspring with limited incidence of inherited diseases [5]. However, the accuracy of COI in determining the loss of genetic diversity depends on their depth [6, 7]. To accurately determine the genetic diversity retained in any one animal, pedigrees will need to go back to the founder population for the dog breed [6, 7]. Even if it was possible for pedigrees to reach the founding population, the inbreeding that occurs during development of the breed prior to registration is usually unclear and often widely debated. For some recently developed dog breeds this may not be an issue, but for breeds like the golden retriever (GR) COIs would likely need to be based upon >20 generations.

A resolution to the issue of COI percentages that lack depth is utilization of modern genetic techniques involving DNA analysis. To date, the two methods available to evaluate the genetic contribution of the founding population to a dog is via short-tandem repeat (STR) markers and single nucleotide polymorphisms (SNPs) [8–14]. It was previously estimated that most modern dog breeds retain 87% of the diversity present in the founders [15]. Therefore, both STR and SNPs can provide a more accurate assessment of the genetic contribution of founders that remains in contemporary members of the breed.

With regard to STR markers, they are contiguous repeated segments of DNA consisting of two or more nucleotides. In humans, forensic DNA analysis utilizes 10–15 STRs to identify individuals, illustrating their utility for genetic profiling [16]. More recently, they have been adopted by the UC Davis Veterinary Genetics Laboratory (UCD VGL) to evaluate a dog’s heterogeneity, allelic richness, genetic drift and diversity [9–11]. SNPs have also emerged as a strong tool to evaluate the heterogeneity and diversity of a dog [12–14]. One advantage of utilizing SNPs instead of STR marker panels is that SNPs can be used to detect previously identified causative mutations associated with disease in dogs being tested [14]. However, there have been numerous studies that illustrate how STRs are better predictors of genetic relatedness than SNPs in both humans and non-human primates [17, 18]. Genetic analysis using SNPs also requires higher yields of DNA to optimize DNA probe hybridization and requires more computational analysis to evaluate allelic diversity compared to STR marker panels [8–14, 19]. Additionally, utilization of STR panels also allows breeders and researcher to interrogate the highly polymorphic regions of the dog genome such as the dog leukocyte antigen (DLA) [9–11]. Such regions would normally require sequencing of a large number of SNPs to identify all haplotypes. The DLA region is in strong linkage disequilibrium and tends to be inherited relatively intact through many generations. Therefore, it can also represent an important marker for breed founders.

In this manuscript, we chose to utilize two different methods to evaluate GR genetic diversity: an STR marker panel derived internal relatedness (IR) score and pedigree derived 10-generation (10-G) and 12-generation (12-G) COI percentages. The GR breed is an ideal breed for this type of analysis due to the large breed population size, which is assumed to be genetically diverse. Additionally, the GR breed has been subjected to two major artificial selection pressures resulting in two distinct lineage groups: conformation and performance. We used an STR marker panel instead of a SNP panel to assess the genetic heterogeneity and diversity of golden retrievers (GRs) due to logistics, price, and practicality. Furthermore, public pedigree database records are also available to GR breeders that include 10-G and 12-G COI percentages. We hypothesize that breeding practices that result in distinct GR lineage groups impact the overall genetic diversity for this breed.

## Materials and methods

### Sample collection

Pedigreed North American golden retrievers (GRs) were recruited to participate in this study via open enrollment at multiple breed events and website advertisement. Cytology brushes and pedigree information for all participating GRs were collected. A comprehensive questionnaire that included owner-reported GR lineage and demographic data was required for study enrollment. Lineage of the GR was recorded as conformation or performance.

### DNA extraction

Approval from the UC Davis Institutional Animal Care and Use Committee was obtained (Protocol Number: 20047) prior to collection of samples. DNA was extracted from cytology brushes as previously described [8]. Briefly, brush samples were incubated for ten minutes at 95°C in 400 μL of 50nM NaOH followed by the addition of 140 μL of 1M Tris-HCL, at pH 8.0 to neutralize the reaction. An aliquot from the extracted DNA was utilized for genotyping.

### STR and DLA haplotype analysis

To determine the genetic diversity of GRs we utilized 33 STR markers that were previously validated [9–11]. The 33 STR markers consisted of: 19 dinucleotide-STRs and 2 tetranucleotides-STRs recommended by the International Society of Animal Genetics (ISAG) for parentage verification; 10 tetra-STRs validated for forensic testing as well as markers for the amelogenin (*AMELX and AMELY*) genes for sex determination [20–22]. The STRs were run on four panels at the UCD VGL.

STR results were used to calculate an IR score for each dog using the following formula: *IR = (2H-Σf*_*i*_*)/(2N-Σf*_*i*_*)* where H corresponds to the number of loci that are homozygous for an individual, N is the total number of loci genotyped, and f_i_ is the frequency of the i-th allele [23]. After, IR values were adjusted to take into consideration accumulated GR inbreeding. This is achieved by adjusting the alleles and allele frequencies observed in the GR breed with the same alleles and allele frequencies observed in village dogs. An IR score evaluates the homozygosity of each STR locus putting more weight on identified rare alleles. An IR value can range from -1 to 1. Our cutoff to define inbreeding was based on the UCD VGL analysis of unrelated Standard Poodles that used Illumina 170k Canine HD BeadChip (Illumina, San Diego CA) genotyping to confirm the power of the STR panel to evaluate genetic relatedness [24]. This analysis determined that dogs were unrelated when the IR score were ≤ 0.15 [24]. Additionally, a full-sibling mating will result in an average value of 0.25 and GRs with an IR score >0.25 correspond to an offspring from a highly-inbred sire and dam combination.

To determine DLA class I and II haplotypes, we utilized the STRs flanking these regions as previously described and validated [9–11]. Linkage between DLA class I and II haplotypes was determined using four and three flanking STRs for DLA class I and II using PHASE v2.1 analysis [25].

Finally, to analyze the number of distinct STR and DLA markers for the GR breed, we obtained the number of overall alleles reported for 17 breeds tested to date by the UCD VGL.

### COI percentage

Both a GR 10-G and 12-G COI percentage were obtained from the GR public pedigree database website k9data.com. This percentage is calculated from pedigree analysis and is a measure of shared ancestors. Briefly, COI percentages are calculated based on the sum of the paths in a pedigree with the following formula: *f = (1/2)*^*i-1*^ *x (1+F*_*CA*_*)* [26]. A path refers to a loop in a pedigree that is required to go from a common ancestor to the parents of an individual dog without any repeated ancestors [26]. In the formula *i* is the number of individuals in the path and F_CA_ is the inbreeding coefficient of the shared ancestor [26]. We selected a 10% COI as our cutoff to define inbreeding since there are reported reductions in reproductive success as well as litter size and longevity at COI’s ≥10% [27, 28]. Additionally, a 12.5% COI is indicative of breeding half-siblings or a grandparent and grandpup mating and COI percentages >25% are a result of mating a parent with their offspring and/or full-sibling breeding [28].

### Statistical analysis

Descriptive statistics were conducted for COI percentages and IR scores to evaluate diversity of the overall population and each lineage group. D’Agostino-Pearson normality test was utilized to determine if COI percentages and IR scores were normally distributed. If the data is normally distributed, we reported the mean +/- SD and for nonparametric data median and interquartile range (IQR) was reported. A Mann-Whitney test was conducted to evaluate if IR scores and COI percentages differed significantly by lineage. Spearman nonparametric correlation analysis was used to evaluate correlations between IR score and 10-G or 12-G COI percentage. GraphPad Prism v7 (GraphPad Software INC. La Jolla, CA) was used for all statistical analysis performed in this study.

We further evaluated the 33 STR markers allele frequencies and seven DLA class I and II STR loci associated allele frequencies using GenAlEx 6.5 [29]. This allowed determination of allelic richness (Na), observed heterozygosity (Ho) and the fixation index (F) for the entire GR sample population dataset as well as individual purpose and lineage groups. Furthermore, principal coordinate analysis (PCoA) of IR scores was performed using GenAlEx 6.5 [29].

## Results

### Sample population

**Fig 1:**
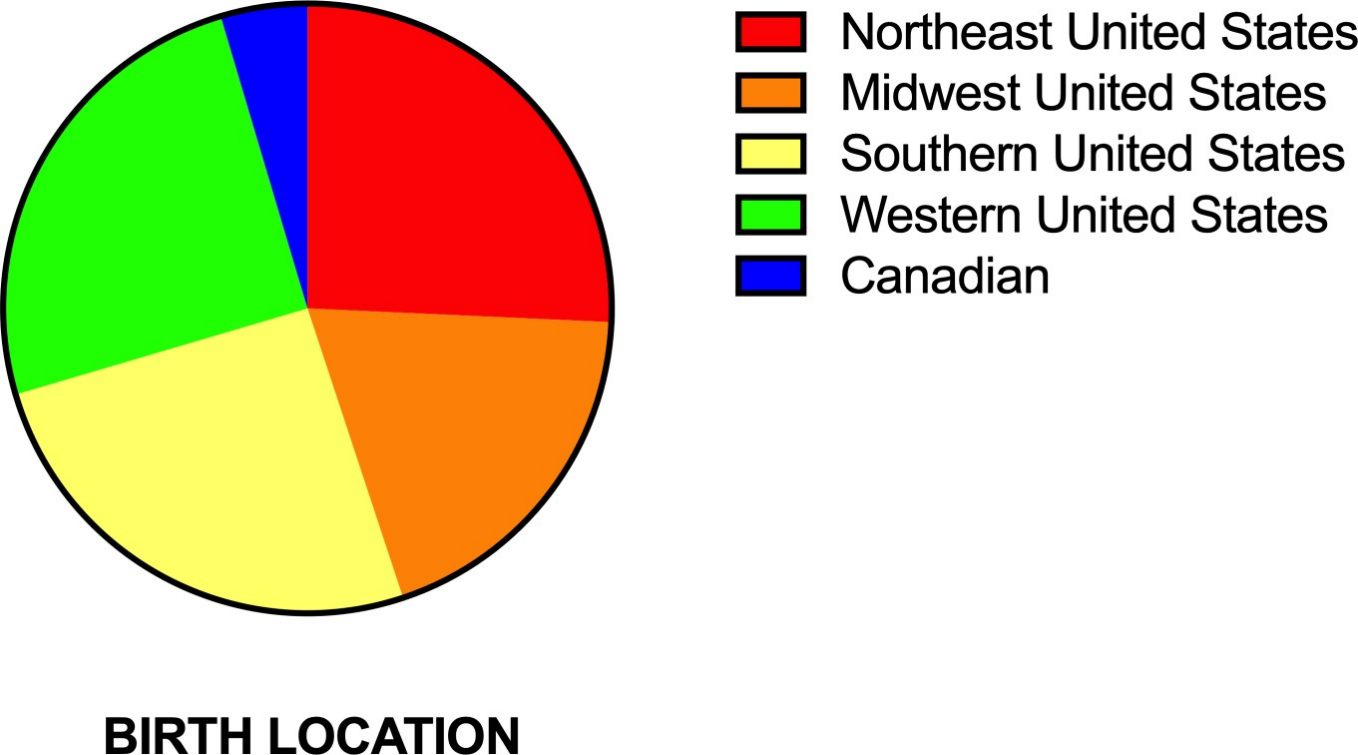
Sample population demographics grouped by Census Bureau-designated regions are presented in pie chart form to depict the variation in birth location.

A total of 529 GRs from across North America were recruited to participate in this study. We had equal representation of GRs from all geographical regions of the United States (Fig 1). Additionally, we had Canadian GRs that contained American Kennel Cub (AKC) registered GRs in their pedigree. Four samples did not yield appropriate quality DNA and were thus excluded, yielding a total of 525 GR samples for analysis. Of these 525 samples, median age was 4.79 years (Range: 0.17–16.3 years) and included 278 females and 247 males. Data on reported dog lineage was available for 386 dogs. The conformation lineage group contained a total of 297 dogs (128 male and 169 female) and had a median age of 4.9 years (Range: 0.17–15 years). The performance group contained a total of 89 dogs (48 male and 41 female) and had a median age of 4.7 years (Range: 0.93 to 16 years).

### IR score analysis by lineage

We determined the allele frequencies for 133 STR markers in the 525 GR samples enrolled in this study (S1 Table). Overall, the number of identified alleles per loci ranged from 3 to 13 alleles and the percent from 22% to 70% (mean 46% of known canid diversity). The GR breed contained the lowest percent of known alleles for STR loci AHTH130, INU030, and REN247M23 (22%, 23%, and 25% respectively) and the highest percent of known alleles for STR loci VGL2009 and VGL2409 (70%) (S1 Table). We observed a higher number of effective alleles (Ne) in performance GRs than the conformation GRs (Ne = 3.54 and 3.14 respectively) (Table 1). Golden Retrievers from performance lines also had a lower fixation index compared to the conformation lineage GRs. All other genetic parameters analyzed were similar between both lineage groups. Additionally, heterozygosity analysis for the individual STR loci, identified that the performance GRs had more loci with negative fixation index values compared to conformation GRs (8 and 6 respectively) and overall the GR breed only contained five STR loci with a negative fixation index (S2 Table).

**Table 1:** Genetic assessment of 33 short tandem repeat (STR) markers for lineage groups.

Lineage Group		Na	Ne	Ho	He	F
Overall (386)	Mean	6.212	3.342	0.651	0.675	0.035
	SE	0.252	0.133	0.012	0.011	0.008
Conformation (297)	Mean	6.394	3.141	0.631	0.656	0.041
	SE	0.364	0.174	0.044	0.016	0.009
Performance (89)	Mean	6.030	3.542	0.671	0.693	0.030
	SE	0.352	0.197	0.017	0.015	0.013

Table headers correspond to the following: Na = number of different alleles, Ne = number of effective alleles, Ho = observed heterozygosity, He = expected heterozygosity, and F = fixation index.

**Fig 2:**
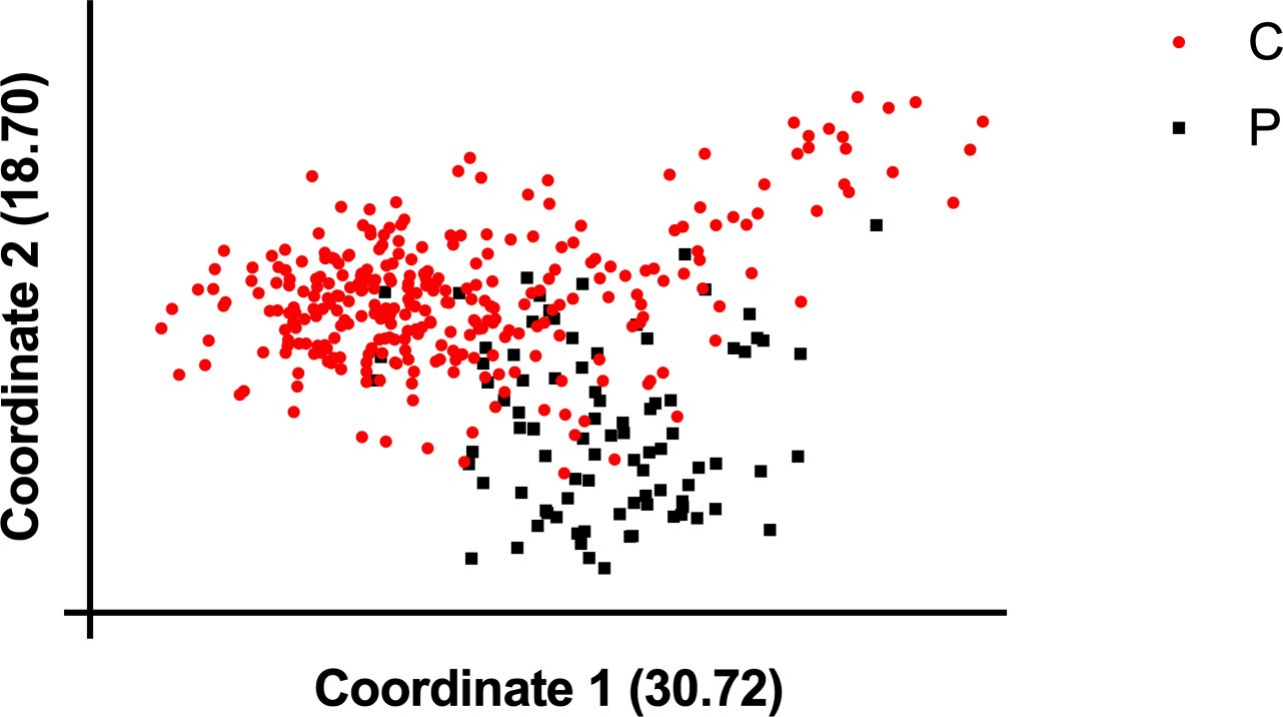
Principal coordinate analysis (PCoA) plot illustrating the genetic relationship between 297 conformation and 89 performance Golden Retrievers.

To determine the genetic relationship between conformation and performance lines, we performed PCoA using the 33 STR markers. Overall, the GR population formed one cluster with considerable diversity as indicated by genetic outliers (Fig 2). There was also noticeable population substructure between conformation and performance lines.

**Fig 3:**
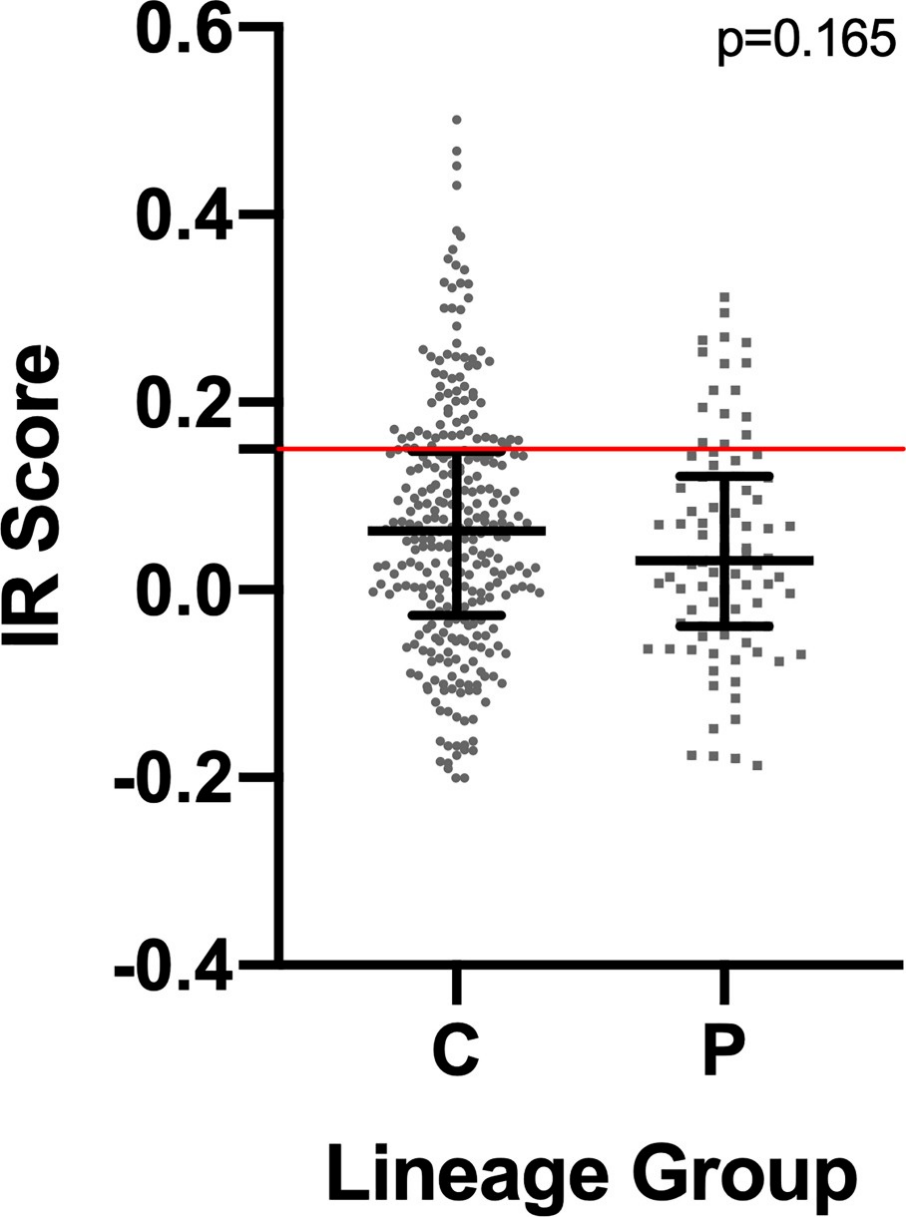
Mann-Whitney test analysis results evaluating internal relatedness (IR) scores for Golden Retrievers grouped by lineage. Red line denotes the IR cutoff score that defines inbreeding (0.15). The figure legend letters correspond to the following lineage groups: C = conformation and P = performance.

Allele frequencies for 33 STR markers were used to calculate IR scores for individual dogs in the entire population. The median IR scores ranged -0.266 (most heterogenous) to 0.501 (least heterogenous). The median for the population was 0.049 (IQR: -0.040 to 0.143), for conformation lines the median was 0.063 (IQR: -0.027 to 0.15), and for performance lines the median was 0.032 (IQR: -0.039 to 0.12). We did not identify any statistically significant differences for IR between the conformation and performance lines (p = 0.165) (Fig 3).

### DLA class I and II haplotype analysis

We next evaluated DLA class I and II STR haplotypes for the 522 of the 525 GRs and identified a total of 21 class I and 20 class II haplotypes in the GR breed. The GR possessed only 12% and 22% of the known canid class I and class II DLA haplotypes, respectively. The majority of class I haplotypes identified in the GR were shared among all dog breeds tested, except for DLA class I 1069 which was unique to the GR and found in 3% individuals (S3 Table). DLA class II haplotypes 2045, 2047, 2051, and 2058 were also unique to GRs (S3 Table). The greatest DLA class I and DLA class II sharing were with Labrador Retriever and Havanese breeds.

We identified 14 DLA class I and 16 DLA class II haplotypes for GRs grouped by lineage (Table 2). The conformation group contained the least number of haplotypes for both DLA class I and class II when compared to performance GRs. The DLA class I haplotypes 1065 and 1066 were the most prevalent in both lines (26% and 28%, respectively), while DLA class I haplotype 1065 was the most common haplotype in conformation lines and 1066 the most common in performance lines. The DLA class II haplotypes 2046 and 2048 were identified in 26% and 25% of the GRs, respectively, with 2046 more common in the performance lineage and 2048 in the conformation lineage (Table 2). We further evaluated the DLA class I and II allele frequency to determine the current heterozygosity and found fixation index results for performance GRs of -0.009 and for conformation GRs of 0.012 (Table 3).

**Table 2:** Dog leukocyte antigen (DLA) class I and II allele frequencies for 386 Golden Retrievers grouped by lineage.

DLA Class	STR Haplotype ID	Lineage Group		
		Overall	C	P
	N	386	297	89
Class I	1003	0.171	0.215	0.023
	1006	0.012	-	0.051
	1011	0.001	-	0.006
	1014	0.044	0.037	0.068
	1016	0.001	-	0.006
	1062	0.106	0.104	0.114
	1065	0.262	0.300	0.136
	1066	0.275	0.254	0.347
	1067	0.039	0.019	0.108
	1068	0.051	0.054	0.040
	1069	0.023	0.008	0.074
	1070	0.009	0.005	0.023
	1121	0.001	0.002	-
	1146	0.003	0.002	0.006
Class II	2001	0.171	0.215	0.023
	2003	0.023	0.024	0.023
	2005	0.009	0.005	0.023
	2007	0.012	-	0.051
	2012	0.001	-	0.006
	2017	0.026	0.019	0.051
	2021	0.108	0.106	0.114
	2045	0.023	0.008	0.074
	2046	0.265	0.253	0.307
	2047	0.016	0.015	0.017
	2048	0.256	0.285	0.159
	2050	0.044	0.037	0.068
	2051	0.014	-	0.063
	2052	0.003	0.002	0.006
	2053	0.027	0.030	0.017

Table column header letters correspond to the following lineage groups: C = conformation, and P = performance.

**Table 3:** Genetic assessment of dog leukocyte antigen (DLA) alleles for 386 Golden Retriever samples grouped by lineage.

Lineage Group		Na	Ne	Ho	He	F
Overall (n = 386)	Mean	13.00	5.393	0.810	0.811	0.001
	SE	0.816	0.424	0.018	0.014	0.009
Conformation (n = 297)	Mean	12.00	4.738	0.779	0.789	0.012
	SE	1.000	0.118	0.002	0.005	0.004
Performance (n = 89)	Mean	14.00	6.049	0.841	0.834	-0.009
	SE	1.000	0.453	0.000	0.012	0.015

Table headers correspond to the following: Na = number of different alleles, Ne = number of effective alleles, Ho = observed heterozygosity, He = expected heterozygosity, and F = fixation index.

### Evaluation of COI percentage by lineage

**Fig 4:**
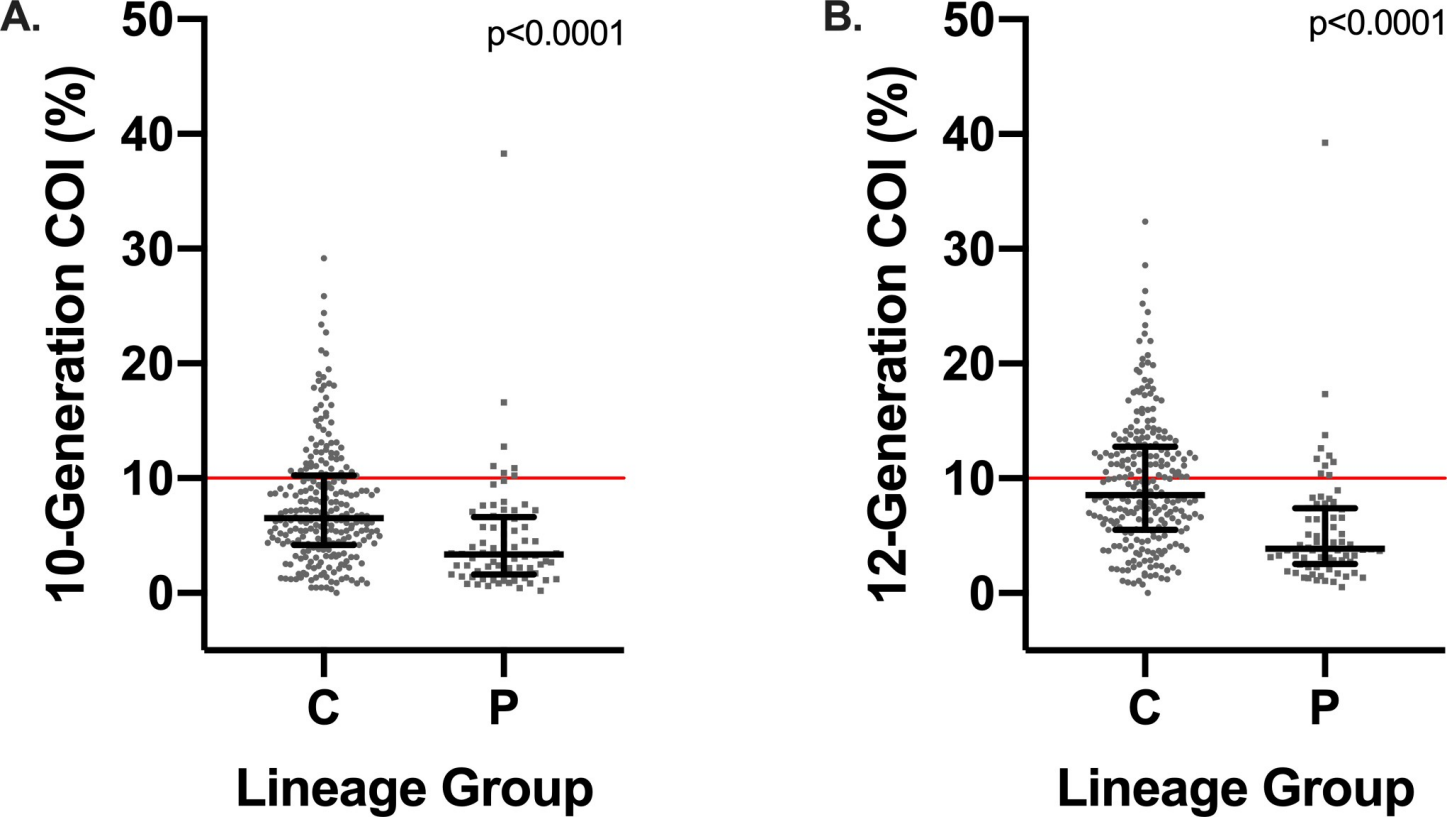
**Statistically significant results for Mann-Whitney test for 10-generation coefficient of inbreeding (COI) percent (A) and 12-generation COI percent (B).** Red line indicates the COI threshold that defines inbreeding (10%). The figure legend letters correspond to the following lineage groups: C = conformation, P = performance.

Three hundred ninety-eight of the 525 GRs in our sample population had COIs calculated from 10-G and 12-G pedigrees. The median 10-G COI for 398 GR samples in this study was 5.82% (IQR: 2.79 to 9.14) and the median 12-G COI was 7.47% (IQR: 3.75 to 11.70) (Table 4). Only 320 GRs had reported lineages and were used for further analysis. The conformation lineage had the highest median 10-G and 12-G COI percentage, while the performance line maintained the lowest COI percentage (Table 4). COIs for both generations were significantly higher (p<0.0001) for conformation lines (Fig 4).

**Table 4:** Median 10-generation, and 12-generation coefficient of inbreeding (COI) percentages for all Golden Retrievers grouped by lineage.

	Overall		Conformation		Performance	
	Median (IQR)	N	Median (IQR)	N	Median (IQR)	N
**10-generation COI**	5.82 (2.79/9.14)	398	6.54 (4.21/10.20)	248	3.35 (1.63/6.64)	72
**12-generation COI**	7.47 (3.75/11.70)	398	8.54 (5.53/12.80)	248	3.89 (2.55/7.40)	72

### Comparison of IR scores and COI percentages

**Fig 5:**
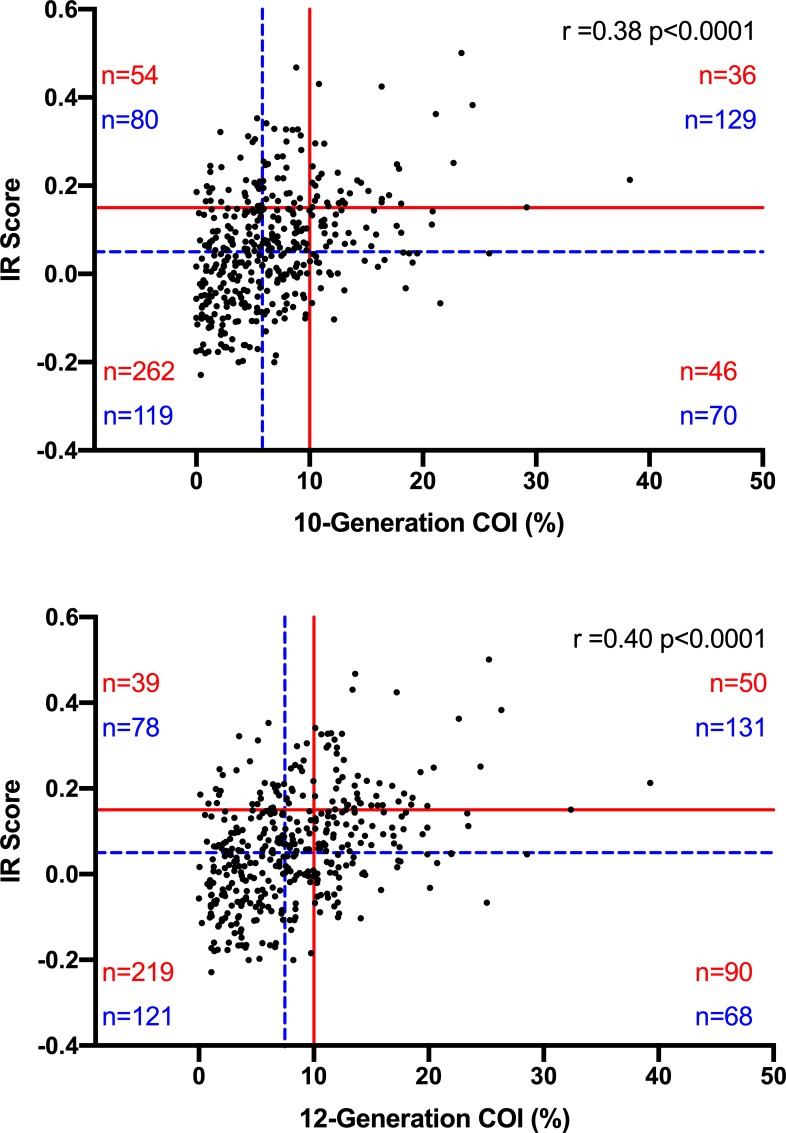
**Spearman correlation analysis results for internal relatedness (IR) score vs. 10-generation (A) and 12-generation (B) coefficient of inbreeding (COI) percentage for 398 Golden Retrievers (GRs).** Solid red lines represent the inbreeding cutoff for IR score (0.15) and COI (10%) percentage, and the dashed blue lines represent the median IR (0.05) score, 10-generation COI (5.82) percentage, and 12-generation COI (7.47) percentage.

**Fig 6:**
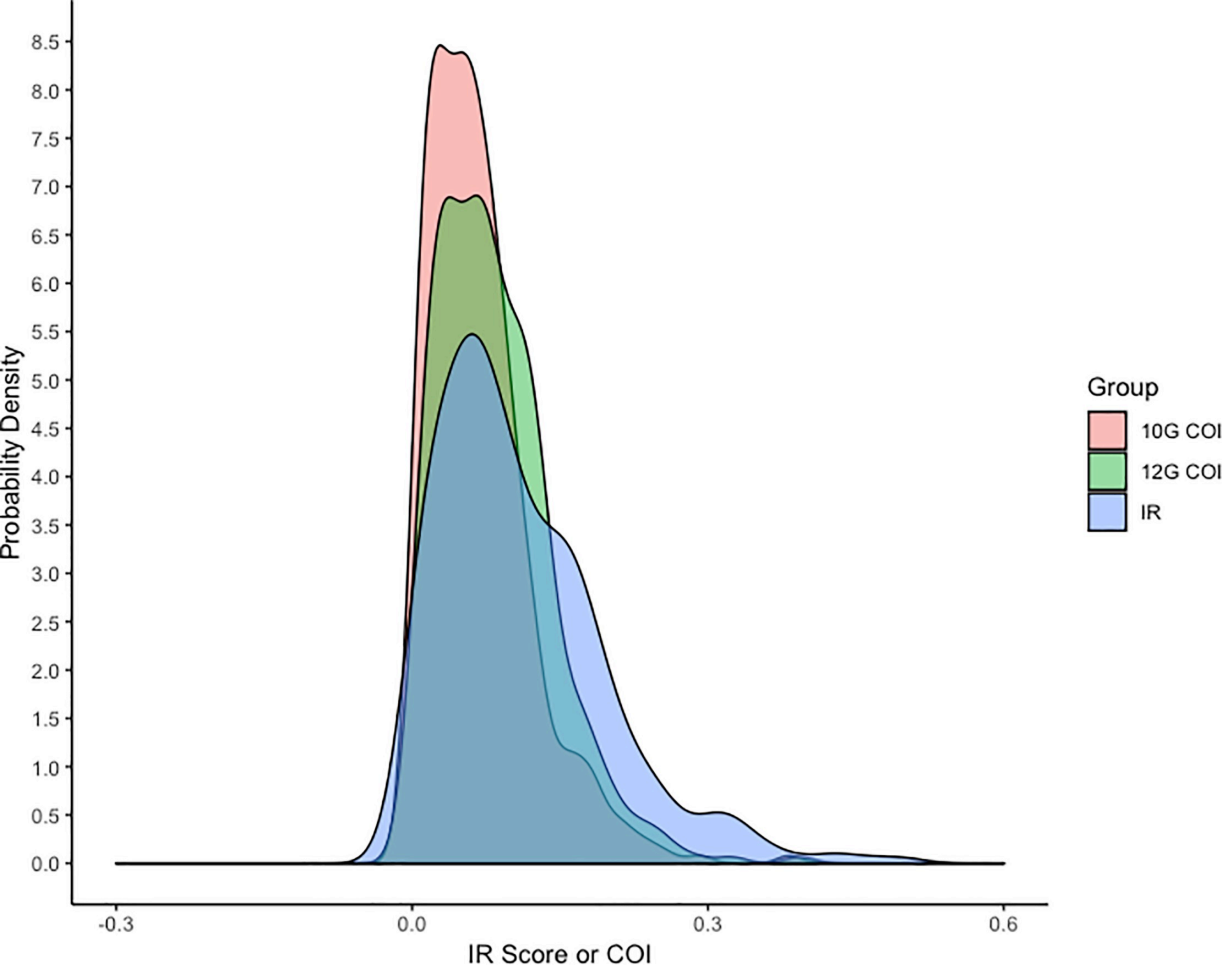
Internal relatedness (IR) sample distribution for 398 GRs is compared to the distribution of 10-generation and 12-generation coefficient of inbreeding (COI) percent displayed as a rational number. The y-axis corresponds to the IR or COI values and the x-axis corresponds to the probability density.

Spearman nonparametric correlation analysis was used to compare IR scores with 10-G or 12-G COIs from the same 398 dogs. The r-values for 10-G and 12-G comparisons were 0.38 and 0.40, respectively (Fig 5). Nine percent of samples had COI and IR scores exceeding the proposed threshold for inbreeding, and 66% of the samples were below. Thirty-two percent of GRs exceeded both the median COI percent and IR score, and 30% of samples were below the median. Thirteen percent of the samples with IR scores and 12-G COIs exceeded the inbreeding threshold and 55% of samples were below. Ten generation COIs had a narrow distribution, while IR scores were distributed over a greater range (Fig 6).

## Discussion

The aim of this manuscript was to illustrate how DNA and 33 autosomal STR loci can be utilized to evaluate the genetic diversity of pure breeds of dogs. We have chosen to model the GR breed because of its popularity and reported heterogeneity. A low density STR marker panel was chosen over a high-density SNP array due to logistics, price, and practicality. Results of this study indicated that the GR breed has retained 46% of the known genetic diversity in all dogs. The average number of alleles per loci and the proportion of alleles contributing to heterozygosity, observed and expected heterozygosity and resulting fixation index, indicated that the breed was in good genetic health. However, IR scores calculated from the same allele frequencies indicated that a proportion of individuals was more inbred and outbred than the average for the population. Such information is important for breeders to better manage genetic diversity in individuals as well as the overall population. A reduction in heterozygosity across the breed or within a subpopulation can decrease health, litter size, and lifespan [28].

DNA testing of this type can also be of value at looking at subpopulations within the breed that have been created by artificial selection. These subpopulations can occur from the use of sire that gain notoriety in areas such as conformation or performance. The evolution of such populations may grow to an extent as to effect genetic diversity within the subpopulation (line) or the entire population. In order to study the effects of such selection we chose to look at GRs bred for conformation or performance. Observed and expected heterozygosity for both groups was similar, illustrating that each group was genetically heterogenous. Fixation index evaluation illustrated that the performance lines were more heterogenous compared to conformation lines. IR scores also tended to be lower in the performance lines. However, the level of significance was only p = 0.17. This result is similar to previous findings that illustrated that conformation lines have reduced heterogeneity and genetic diversity compared to performance lines although both groups have decreased genetic heterogeneity and diversity when compared to village dogs [3].

Evaluation of the DLA haplotypes indicate that GRs had retained 12% and 22% of the respective DLA class I and class II haplotypes known to exist in modern canids. This suggest that the breed has evolved from no more than 16 founder lines, each possessing the existing haplotypes. The confirmation GRs contained less haplotypes compared to performance lines. Furthermore, the two most common haplotypes identified for DLA class I and class II were different between each lineage group. The presence of these haplotypes relates to the artificial selection that were used to select for conformation or performance. This also supports the findings from the 33 autosomal STRs. It is important for breeders to always consider the genetic diversity of the DLA in breed management because polymorphisms within genes of the DLA play a crucial role in autoimmunity [9–11, 30, 31].

COIs calculated from relatively shallow pedigrees have been historically used by breeders to monitor heterozygosity and genetic diversity within their dog breeds[6, 32]. It is presumed that COIs are only of great value when they include the founder population [6,7]. GR breeders based their COIs on 10-G or 12-G pedigrees. Twelve generations are not sufficient to reach the American Kennel Club founder population that goes back to 1932, or approximately 22 generations. The value of including more generations was shown by COI calculations comparing 10 and 12 generations. Both COIs and IR values increased from 10 to 12 generations, COI more so than IR. In the absence of complete generational pedigrees, we would suggest that DNA analysis may be a reasonable surrogate for genetic contributions of founders to contemporary dogs.

## Conclusion

This study demonstrated how DNA analysis based on 33 autosomal STR loci markers can evaluate genetic heterogeneity and diversity in a pure breed such as the GR. We found the 525 GRs tested to be heterogenous by standard genetic assessment and to segregate as a single population by PCoA. Overall, the breed has retained about half of known alleles present in all canids. Although the population appeared heterogenous, IR values indicated that portions of the population were either more inbred or outbred than the average GR. Artificial selection for conformation or performance led to sub-structuring in the population with conformation having a greater effect than performance. Seven additional STR markers within the DLA identified 21 class I and 20 class II haplotypes. This was a small proportion of all known haplotypes and indicative of a low number of breed founders. Additional narrowing of DLA haplotypes was observed in both conformation and performance subpopulations. A weak but significant relationship was found between IR calculated from DNA and from COIs calculated from 10 or 12 generation pedigrees. DNA testing using a low density STR marker panel provided an accurate assessment of genetic heterogeneity, population substructure, and diversity in a model breed such as the GR.

## Supporting information

S1 Table Allele frequency for the 33 short tandem repeat markers in the 525 Golden Retrievers tested. The number of known alleles for all dogs tested by the UC Davis Veterinary Genetics Laboratory are listed under the locus name in parenthesis. The percent of known alleles detected in the Golden Retriever breed are listed in bold at the end of the allele list.
(DOCX)

S2 Table Genetic assessment for 33 short tandem repeat markers for 386 Golden Retrievers grouped by lineage. Loci with increased heterozygosity are in listed in bold letters.
(DOCX)

S3 Table Dog leukocyte antigen (DLA) class I and class II haplotype sharing between Golden Retrievers and other dog breeds tested by the UC Davis Veterinary Genetics Laboratory. Total number of DLA class I and class II haplotypes identified in all dogs are listed in parenthesis.
(DOCX)
