# Supplementary material for: Genetic heterogeneity and diversity of North American golden retrievers using a low density STR marker panel
Source: PLoS One. 2019 Feb 27;14(2):e0212171. doi: 10.1371/journal.pone.0212171 (PMC6392251; doi:10.1371/journal.pone.0212171)
Supplement: S1 Table — The number of known alleles for all dogs tested by the UC Davis Veterinary Genetics Laboratory are listed under the locus name in parenthesis. The percent of known alleles detected in the Golden Retriever breed are listed in bold at the end of the allele list. (DOCX) [file pone.0212171.s001.docx]

| **AHT121** | **AHT137** | **AHTH130** | **AHTh171-A** | **AHTh260** | **AHTk211** |
| --- | --- | --- | --- | --- | --- |
| **(22)** | **(16)** | **(18)** | **(14)** | **(18)** | **(8)** |
| 96 (26.42) | 131 (15.41) | 117 (3.89) | 219 (63.99) | 240 (1.81) | 89 (8.29) |
| 98 (4.53) | 133 (16.84) | 121 (31.87) | 223 (1.81) | 242 (11.66) | 91 (11.27) |
| 100 (0.13) | 137 (15.67) | 125 (36.79) | 225 (19.43) | 244 (61.66) | 93 (65.80) |
| 102 (47.80) | 143 (8.55) | 129 (27.46) | 227 (11.92) | 246 (16.45) | 95 (3.76) |
| 104 (1.68) | 145 (2.07) |  | 237 (2.85) | 248 (6.74) | 97 (10.88) |
| 106 (16.84) | 147 (8.55) |  |  | 250 (0.39) |  |
| 108 (1.55) | 149 (18.13) |  |  | 252 (0.13) |  |
| 110 (0.91) | 151 (10.23) |  |  | 254 (1.17) |  |
| 112 (0.13) | 153 (4.53) |  |  |  |  |
| **41%** | **56%** | **22%** | **36%** | **44%** | **63%** |
| **AHTk253** | **C22.279** | **FH2001** | **FH2054** | **FH2848** | **INRA21** |
| **(11)** | **(13)** | **(16)** | **(15)** | **(14)** | **(11)** |
| 284 (0.26) | 116 (21.63) | 124 (10.62) | 148 (16.71) | 232 (6.97) | 91 (26.04) |
| 286 (32.51) | 118 (54.92) | 132 (17.49) | 152 (1.30) | 234 (5.05) | 95 (7.77) |
| 288 (29.66) | 120 (5.83) | 136 (2.72) | 156 (39.38) | 236 (1.68) | 97 (0.78) |
| 290 (0.52) | 124 (4.92) | 140 (5.05) | 160 (3.89) | 238 (45.08) | 99 (6.87) |
| 292 (37.05) | 126 (12.69) | 144 (36.01) | 164 (1.30) | 240 (41.19) | 101 (29.02) |
|  |  | 148 (17.88) | 168 (24.87) | 242 (0.13) | 103 (29.53) |
|  |  | 152 (8.94) | 172 (11.14) |  |  |
|  |  | 156 (1.17) | 176 (1.30) |  |  |
|  |  | 160 (0.13) | 180 (0.13) |  |  |
| **46%** | **39%** | **57%** | **60%** | **43%** | **55%** |
| **INU005** | **INU030** | **INU055** | **LEI004** | **REN105L03** | **REN162C04** |
| **(16)** | **(13)** | **(12)** | **(11)** | **(14)** | **(15)** |
| 110 (9.07) | 144 (40.41) | 210 (11.27) | 85 (7.77) | 227 (0.26) | 202 (6.74) |
| 124 (22.02) | 150 (51.04) | 214 (46.37) | 95 (34.59) | 231 (6.35) | 204 (36.79) |
| 126 (54.92) | 152 (8.55) | 216 (27.20) | 97 (9.33) | 233 (60.23) | 206(30.44) |
| 128 (13.73) |  | 218 (15.16) | 107 (48.32) | 235 (21.50) | 208(26.04) |
| 132 (0.26) |  |  |  | 237 (0.65) |  |
|  |  |  |  | 239 (3.24) |  |
|  |  |  |  | 241 (7.77) |  |
| **32%** | **23%** | **33%** | **36%** | **50%** | **27%** |
| **REN169D01** | **REN169O18** | **REN247M23** | **REN54P11** | **REN64E19** | **VGL0760** |
| **(15)** | **(13)** | **(12)** | **(14)** | **(12)** | **(20)** |
| 202 (34.46) | 162 (30.44) | 268 (37.05) | 222 (4.92) | 139 (3.37) | 14 (0.13) |
| 212 (12.82) | 164 (39.38) | 272 (38.21) | 226 (2.85) | 143 (0.78) | 18.2 (0.52) |
| 206 (41.19) | 168 (10.36) | 278 (24.74) | 228 (1.55) | 145 (26.30) | 19.2 (16.45) |
| 218 (9.72) | 170(18.52) |  | 232 (34.59) | 147 (37.56) | 20.2 (17.62) |
| 220 (1.81) | 172 (1.30) |  | 234 (42.62) | 149 (17.88) | 21.2 (36.01) |
|  |  |  | 236 (10.23) | 153 (14.12) | 22.2 (9.97) |
|  |  |  | 238 (3.28) |  | 23.2 (16.32) |
|  |  |  |  |  | 24.2 (2.33) |
|  |  |  |  |  | 25.2 (0.65) |
| **33%** | **39%** | **25%** | **50%** | **50%** | **45%** |
| **VGL0910** | **VGL1063** | **VGL1165** | **VGL1828** | **VGL2009** | **VGL2409** |
| **(25)** | **(17)** | **(24)** | **(14)** | **(10)** | **(10)** |
| 14 (1.30) | 8 (0.65) | 14 (0.13) | 14 (12.69) | 11 (0.78) | 14 (19.56) |
| 15 (1.04) | 12 (1.55) | 16 (11.01) | 15 (9.97) | 12 (15.41) | 15 (8.03) |
| 15.1 (1.42) | 13 (59.97) | 17 (23.96) | 16 (1.81) | 13 (18.01) | 16 (38.08) |
| 16 (0.52) | 14 (3.76) | 18 (0.78) | 19 (39.38) | 14 (20.98) | 17 (30.31) |
| 17.1 (4.27) | 15 (0.13) | 19 (14.51) | 20 (11.01) | 15 (39.12) | 18 (1.94) |
| 18.1 (51.8) | 17 (0.13) | 20 (0.26) | 21 (1.04) | 16 (5.57) | 19 (1.94) |
| 19 (0.39) | 18 (24.35) | 21 (1.81) | 22 (23.70) | 17 (0.13) | 20 (0.13) |
| 19.1 (28.5) | 19 (8.68) | 26 (0.13) | 23 (0.39) |  |  |
| 20 (0.52) | 20 (0.78) | 27 (8.81) |  |  |  |
| 20.1 (2.20) |  | 28 (32.38) |  |  |  |
| 21 (0.13) |  | 29 (1.04) |  |  |  |
| 21.1 (7.25) |  | 30 (4.92) |  |  |  |
| 22.1 (0.65) |  | 31 (0.26) |  |  |  |
| **52%** | **53%** | **54%** | **57%** | **70%** | **70%** |
| **VGL2918** | **VGL3008** | **VGL3235** |  |  |  |
| **(21)** | **(17)** | **(16)** |  |  |  |
| 12 (27.85) | 13 (0.13) | 12 (42.75) |  |  |  |
| 13 (11.92) | 14 (45.98) | 13 (0.39) |  |  |  |
| 14 (0.39) | 15 (0.39) | 14 (5.18) |  |  |  |
| 17.3 (1.55) | 18 (1.94) | 15 (44.17) |  |  |  |
| 18.3 (0.52) | 19 (22.54) | 16 (2.98) |  |  |  |
| 19.3 (52.7) | 20 (4.27) | 17 (2.20) |  |  |  |
| 20.3 (5.05) | 21 (7.25) | 18 (2.33) |  |  |  |
|  | 22 (5.96) |  |  |  |  |
|  | 23 (6.87) |  |  |  |  |
|  | 24 (4.40) |  |  |  |  |
|  | 25 (0.26) |  |  |  |  |
| **33%** | **65%** | **44%** |  |  |  |
