# Supplementary material for: Genetic heterogeneity and diversity of North American golden retrievers using a low density STR marker panel
Source: PLoS One. 2019 Feb 27;14(2):e0212171. doi: 10.1371/journal.pone.0212171 (PMC6392251; doi:10.1371/journal.pone.0212171)
Supplement: S2 Table — Loci with increased heterozygosity are in listed in bold letters. (DOCX) [file pone.0212171.s002.docx]

| Population | STR Locus | N | Na | Ne | Ho | He | F |
| --- | --- | --- | --- | --- | --- | --- | --- |
| Overall | AHT121 | 22.0 | 8.00 | 3.19 | 0.67 | 0.68 | 0.03 |
|  | AHT137 | 16.0 | 9.00 | 7.26 | 0.83 | 0.86 | 0.04 |
|  | AHTH130 | 18.0 | 4.00 | 2.96 | 0.63 | 0.66 | 0.05 |
|  | AHTh171-A | 14.0 | 5.00 | 2.47 | 0.52 | 0.56 | 0.06 |
|  | AHTh260 | 18.0 | 7.50 | 2.57 | 0.54 | 0.59 | 0.08 |
|  | **AHTk211** | **8.00** | **5.00** | **2.10** | **0.55** | **0.52** | **-0.06** |
|  | **AHTk253** | **11.0** | **4.00** | **3.00** | **0.68** | **0.67** | **-0.02** |
|  | C22.279 | 13.0 | 5.00 | 2.87 | 0.64 | 0.65 | 0.02 |
|  | FH2001 | 16.0 | 8.00 | 4.33 | 0.73 | 0.77 | 0.05 |
|  | FH2054 | 15.0 | 8.50 | 4.24 | 0.74 | 0.76 | 0.02 |
|  | FH2848 | 14.0 | 5.50 | 2.72 | 0.57 | 0.63 | 0.09 |
|  | **INRA21** | **11.0** | **6.00** | **4.05** | **0.79** | **0.75** | **-0.05** |
|  | INU005 | 16.0 | 4.50 | 2.57 | 0.60 | 0.61 | 0.02 |
|  | INU030 | 13.0 | 3.00 | 2.26 | 0.52 | 0.56 | 0.07 |
|  | INU055 | 12.0 | 4.00 | 3.14 | 0.66 | 0.68 | 0.03 |
|  | LEI004 | 11.0 | 4.00 | 2.51 | 0.58 | 0.60 | 0.03 |
|  | REN105L03 | 14.0 | 6.00 | 2.66 | 0.57 | 0.61 | 0.07 |
|  | REN162C04 | 15.0 | 4.00 | 3.17 | 0.59 | 0.68 | 0.14 |
|  | REN169D01 | 15.0 | 5.00 | 3.16 | 0.64 | 0.68 | 0.07 |
|  | **REN169O18** | **13.0** | **5.00** | **3.23** | **0.70** | **0.69** | **-0.01** |
|  | REN247M23 | 12.0 | 3.00 | 2.73 | 0.63 | 0.63 | 0.00 |
|  | REN54P11 | 14.0 | 7.00 | 3.28 | 0.66 | 0.69 | 0.05 |
|  | REN64E19 | 12.0 | 6.00 | 3.69 | 0.68 | 0.73 | 0.06 |
|  | VGL0760 | 20.0 | 7.50 | 4.32 | 0.76 | 0.77 | 0.01 |
|  | VGL0910 | 25.0 | 9.00 | 3.27 | 0.68 | 0.67 | 0.00 |
|  | VGL1063 | 17.0 | 6.50 | 2.18 | 0.51 | 0.53 | 0.04 |
|  | VGL1165 | 24.0 | 11.00 | 4.85 | 0.77 | 0.79 | 0.03 |
|  | VGL1828 | 14.0 | 7.50 | 4.25 | 0.70 | 0.76 | 0.07 |
|  | VGL2009 | 10.0 | 6.00 | 3.86 | 0.72 | 0.74 | 0.03 |
|  | VGL2409 | 10.0 | 6.50 | 3.86 | 0.71 | 0.73 | 0.04 |
|  | **VGL2918** | **21.0** | **7.00** | **2.75** | **0.65** | **0.63** | **-0.03** |
|  | VGL3008 | 17.0 | 10.00 | 4.09 | 0.70 | 0.74 | 0.05 |
|  | VGL3235 | 16.0 | 7.00 | 2.70 | 0.56 | 0.63 | 0.10 |
| Conformation | AHT121 | 22.0 | 8.00 | 2.91 | 0.61 | 0.66 | 0.07 |
|  | AHT137 | 16.0 | 9.00 | 6.96 | 0.83 | 0.86 | 0.03 |
|  | AHTH130 | 18.0 | 4.00 | 3.07 | 0.67 | 0.67 | 0.01 |
|  | **AHTh171-A** | **14.0** | **5.00** | **1.80** | **0.45** | **0.44** | **-0.02** |
|  | AHTh260 | 18.0 | 8.00 | 1.93 | 0.40 | 0.48 | 0.16 |
|  | **AHTk211** | **8.00** | **5.00** | **2.17** | **0.55** | **0.54** | **-0.01** |
|  | AHTk253 | 11.0 | 4.00 | 2.95 | 0.66 | 0.66 | 0.01 |
|  | C22.279 | 13.0 | 5.00 | 2.48 | 0.52 | 0.60 | 0.13 |
|  | FH2001 | 16.0 | 8.00 | 4.88 | 0.77 | 0.79 | 0.03 |
|  | FH2054 | 15.0 | 9.00 | 3.47 | 0.71 | 0.71 | 0.00 |
|  | FH2848 | 14.0 | 6.00 | 2.50 | 0.54 | 0.60 | 0.11 |
|  | **INRA21** | **11.0** | **6.00** | **3.89** | **0.76** | **0.74** | **-0.03** |
|  | INU005 | 16.0 | 5.00 | 2.67 | 0.61 | 0.63 | 0.02 |
|  | INU030 | 13.0 | 3.00 | 2.32 | 0.49 | 0.57 | 0.13 |
|  | INU055 | 12.0 | 4.00 | 2.91 | 0.62 | 0.66 | 0.06 |
|  | LEI004 | 11.0 | 4.00 | 2.67 | 0.61 | 0.62 | 0.03 |
|  | REN105L03 | 14.0 | 6.00 | 2.17 | 0.50 | 0.54 | 0.07 |
|  | REN162C04 | 15.0 | 4.00 | 3.34 | 0.68 | 0.70 | 0.03 |
|  | REN169D01 | 15.0 | 5.00 | 3.18 | 0.68 | 0.69 | 0.01 |
|  | **REN169O18** | **13.0** | **5.00** | **3.29** | **0.73** | **0.70** | **-0.05** |
|  | REN247M23 | 12.0 | 3.00 | 2.97 | 0.61 | 0.66 | 0.08 |
|  | REN54P11 | 14.0 | 7.00 | 2.98 | 0.61 | 0.66 | 0.09 |
|  | REN64E19 | 12.0 | 6.00 | 3.71 | 0.68 | 0.73 | 0.06 |
|  | **VGL0760** | **20.0** | **8.00** | **4.38** | **0.79** | **0.77** | **-0.03** |
|  | VGL0910 | 25.0 | 9.00 | 2.43 | 0.56 | 0.59 | 0.05 |
|  | VGL1063 | 17.0 | 9.00 | 2.49 | 0.57 | 0.60 | 0.04 |
|  | VGL1165 | 24.0 | 11.00 | 4.63 | 0.75 | 0.78 | 0.05 |
|  | VGL1828 | 14.0 | 8.00 | 3.64 | 0.66 | 0.73 | 0.09 |
|  | VGL2009 | 10.0 | 7.00 | 3.61 | 0.70 | 0.72 | 0.04 |
|  | VGL2409 | 10.0 | 6.00 | 3.27 | 0.66 | 0.69 | 0.05 |
|  | **VGL2918** | **21.0** | **7.00** | **2.51** | **0.64** | **0.60** | **-0.06** |
|  | VGL3008 | 17.0 | 10.00 | 3.04 | 0.66 | 0.67 | 0.02 |
|  | VGL3235 | 16.0 | 7.00 | 2.43 | 0.55 | 0.59 | 0.07 |
| Performance | **AHT121** | **22.0** | **8.00** | **3.47** | **0.72** | **0.71** | **-0.01** |
|  | AHT137 | 16.0 | 9.00 | 7.57 | 0.83 | 0.87 | 0.04 |
|  | AHTH130 | 18.0 | 4.00 | 2.86 | 0.60 | 0.65 | 0.08 |
|  | AHTh171-A | 14.0 | 5.00 | 3.14 | 0.58 | 0.68 | 0.14 |
|  | AHTh260 | 18.0 | 7.00 | 3.21 | 0.69 | 0.69 | 0.00 |
|  | **AHTk211** | **8.00** | **5.00** | **2.02** | **0.56** | **0.51** | **-0.11** |
|  | **AHTk253** | **11.0** | **4.00** | **3.04** | **0.70** | **0.67** | **-0.04** |
|  | **C22.279** | **13.0** | **5.00** | **3.27** | **0.75** | **0.69** | **-0.09** |
|  | FH2001 | 16.0 | 8.00 | 3.78 | 0.69 | 0.74 | 0.07 |
|  | FH2054 | 15.0 | 8.00 | 5.00 | 0.76 | 0.80 | 0.04 |
|  | FH2848 | 14.0 | 5.00 | 2.94 | 0.61 | 0.66 | 0.08 |
|  | **INRA21** | **11.0** | **6.00** | **4.21** | **0.82** | **0.76** | **-0.08** |
|  | INU005 | 16.0 | 4.00 | 2.46 | 0.58 | 0.59 | 0.02 |
|  | INU030 | 13.0 | 3.00 | 2.19 | 0.54 | 0.54 | 0.01 |
|  | **INU055** | **12.0** | **4.00** | **3.36** | **0.71** | **0.70** | **-0.01** |
|  | LEI004 | 11.0 | 4.00 | 2.35 | 0.56 | 0.57 | 0.02 |
|  | REN105L03 | 14.0 | 6.00 | 3.14 | 0.64 | 0.68 | 0.06 |
|  | REN162C04 | 15.0 | 4.00 | 3.00 | 0.51 | 0.67 | 0.24 |
|  | REN169D01 | 15.0 | 5.00 | 3.15 | 0.60 | 0.68 | 0.13 |
|  | REN169O18 | 13.0 | 5.00 | 3.18 | 0.66 | 0.69 | 0.03 |
|  | **REN247M23** | **12.0** | **3.00** | **2.50** | **0.65** | **0.60** | **-0.09** |
|  | REN54P11 | 14.0 | 7.00 | 3.58 | 0.71 | 0.72 | 0.02 |
|  | REN64E19 | 12.0 | 6.00 | 3.66 | 0.69 | 0.73 | 0.06 |
|  | VGL0760 | 20.0 | 7.00 | 4.26 | 0.73 | 0.76 | 0.05 |
|  | **VGL0910** | **25.0** | **9.00** | **4.10** | **0.80** | **0.76** | **-0.05** |
|  | VGL1063 | 17.0 | 4.00 | 1.87 | 0.45 | 0.47 | 0.04 |
|  | VGL1165 | 24.0 | 11.00 | 5.06 | 0.80 | 0.80 | 0.01 |
|  | VGL1828 | 14.0 | 7.00 | 4.87 | 0.75 | 0.79 | 0.05 |
|  | VGL2009 | 10.0 | 5.00 | 4.10 | 0.74 | 0.76 | 0.02 |
|  | VGL2409 | 10.0 | 7.00 | 4.45 | 0.75 | 0.78 | 0.03 |
|  | VGL2918 | 21.0 | 7.00 | 3.00 | 0.66 | 0.67 | 0.01 |
|  | VGL3008 | 17.0 | 10.00 | 5.14 | 0.74 | 0.81 | 0.08 |
|  | VGL3235 | 16.0 | 7.00 | 2.96 | 0.57 | 0.66 | 0.14 |
