# Supplementary material for: Genetic heterogeneity and diversity of North American golden retrievers using a low density STR marker panel
Source: PLoS One. 2019 Feb 27;14(2):e0212171. doi: 10.1371/journal.pone.0212171 (PMC6392251; doi:10.1371/journal.pone.0212171)
Supplement: S3 Table — Total number of DLA class I and class II haplotypes identified in all dogs are listed in parenthesis. (DOCX) [file pone.0212171.s003.docx]

| **DLA** | **STR ID** | **GR** | **BRT** | **LR** | **DP** | **FCR** | **Hav** | **Sam** | **GS** | **EB** | **Bie** | **IG** | **AKK** | **SS** | **JA** | **MP** | **SV** | **PD** | **TPD** |
| --- | --- | --- | --- | --- | --- | --- | --- | --- | --- | --- | --- | --- | --- | --- | --- | --- | --- | --- | --- |
|  | n | 522 | 124 | 150 | 490 | 419 | 392 | 187 | 186 | 163 | 110 | 764 | 482 | 146 | 330 | 254 | 175 | 2376 | 121 |
| Class I (n=176) | 1003 | 0.154 | -- | 0.003 | -- | -- | 0.034 | -- | -- | -- | -- | -- | -- | -- | -- | 0.010 | -- | 0.168 | -- |
|  | 1006 | 0.012 | 0.040 | 0.037 | -- | -- | 0.047 | 0.005 | 0.048 | 0.003 | -- | -- | -- | -- | -- | 0.004 | 0.251 | 0.047 | 0.004 |
|  | 1008 | 0.002 | -- | 0.070 | -- | -- | -- | -- | 0.048 | 0.006 | 0.009 | 0.135 | 0.060 | -- | -- | -- | -- | 0.018 | 0.021 |
|  | 1011 | 0.001 | -- | -- | -- | -- | -- | 0.273 | 0.013 | -- | -- | -- | 0.058 | -- | -- | 0.004 | -- | 0.020 | 0.025 |
|  | 1012 | 0.001 | -- | -- | -- | -- | 0.018 | 0.013 | -- | 0.414 | 0.177 | 0.009 | -- | -- | -- | 0.049 | -- | 0.010 | 0.029 |
|  | 1014 | 0.041 | 0.036 | -- | -- | -- | 0.034 | 0.003 | 0.245 | -- | 0.027 | -- | 0.364 | -- | -- | 0.004 | -- | 0.009 | 0.045 |
|  | 1016 | 0.001 | 0.008 | 0.013 | 0.014 | -- | 0.216 | -- | 0.040 | 0.095 | 0.027 | 0.058 | -- | -- | -- | 0.024 | -- | 0.015 | 0.033 |
|  | 1030 | 0.001 | -- | 0.023 | 0.101 | -- | 0.001 | -- | -- | -- | 0.477 | 0.024 | -- | -- | -- | -- | -- | 0.003 | -- |
|  | 1040 | 0.001 | -- | -- | 0.011 | -- | 0.022 | -- | -- | 0.040 | 0.109 | 0.097 | 0.222 | -- | -- | 0.006 | -- | 0.001 | 0.004 |
|  | 1050 | 0.001 | -- | -- | -- | -- | -- | -- | -- | -- | -- | 0.001 | -- | -- | -- | -- | -- | -- | -- |
|  | 1059 | 0.001 | -- | -- | -- | -- | -- | -- | -- | -- | -- | 0.098 | -- | -- | -- | -- | -- | -- | -- |
|  | 1062 | 0.103 | -- | 0.017 | -- | -- | -- | -- | -- | 0.187 | -- | -- | 0.213 | -- | -- | -- | -- | -- | -- |
|  | 1065 | 0.262 | -- | 0.377 | -- | 0.001 | -- | -- | 0.003 | -- | -- | 0.001 | -- | -- | -- | -- | -- | -- | -- |
|  | 1066 | 0.283 | -- | 0.003 | -- | 0.001 | -- | -- | -- | -- | -- | -- | -- | -- | -- | -- | -- | -- | -- |
|  | 1067 | 0.042 | -- | -- | -- | 0.001 | -- | -- | -- | -- | -- | -- | -- | -- | 0.006 | -- | -- | -- | -- |
|  | 1068 | 0.050 | -- | 0.050 | -- | 0.266 | 0.015 | 0.043 | 0.038 | -- | -- | -- | -- | 0.240 | -- | 0.018 | 0.346 | -- | 0.012 |
|  | **1069** | **0.032** | -- | -- | -- | -- | -- | -- | -- | -- | -- | -- | -- | -- | -- | -- | -- | -- | -- |
|  | 1070 | 0.012 | -- | 0.007 | -- | -- | -- | -- | -- | -- | -- | -- | -- | -- | -- | -- | -- | -- | -- |
|  | 1121 | 0.001 | -- | -- | -- | -- | 0.006 | -- | -- | -- | -- | -- | -- | -- | -- | -- | -- | -- | -- |
|  | 1128 | 0.001 | -- | -- | -- | -- | 0.003 | -- | -- | -- | -- | -- | -- | -- | -- | -- | -- | -- | -- |
|  | 1134 | 0.001 | -- | 0.010 | -- | -- | 0.003 | -- | -- | -- | -- | -- | -- | -- | -- | -- | -- | -- | -- |
| Class II (n=93) | 2001 | 0.154 | -- | 0.003 | -- | -- | 0.040 | -- | 0.008 | -- | -- | -- | -- | 0.007 | -- | 0.016 | -- | 0.607 | 0.008 |
|  | 2003 | 0.023 | -- | 0.020 | -- | 0.134 | 0.223 | 0.013 | 0.038 | 0.598 | 0.223 | 0.007 | -- | 0.010 | -- | 0.504 | -- | 0.089 | 0.438 |
|  | 2005 | 0.013 | 0.016 | 0.060 | -- | 0.414 | 0.003 | -- | 0.011 | 0.015 | 0.023 | -- | -- | -- | -- | -- | -- | 0.022 | 0.004 |
|  | 2007 | 0.012 | 0.040 | 0.040 | -- | -- | 0.051 | 0.005 | 0.048 | -- | -- | -- | 0.016 | -- | -- | 0.002 | 0.257 | 0.016 | 0.004 |
|  | 2012 | 0.001 | -- | -- | -- | -- | 0.005 | -- | 0.013 | -- | -- | -- | 0.062 | -- | 0.002 | 0.063 | -- | 0.004 | 0.058 |
|  | 2017 | 0.033 | -- | -- | -- | 0.001 | 0.009 | -- | -- | 0.215 | -- | 0.220 | -- | 0.390 | 0.006 | -- | -- | 0.003 | 0.004 |
|  | 2021 | 0.103 | -- | 0.017 | -- | -- | 0.001 | -- | -- | -- | 0.005 | -- | 0.212 | -- | -- | 0.063 | -- | 0.002 | 0.050 |
|  | 2022 | 0.001 | -- | 0.077 | 0.002 | 0.128 | 0.116 | 0.107 | 0.005 | 0.015 | -- | -- | -- | 0.048 | -- | 0.002 | 0.060 | 0.000 | 0.012 |
|  | 2023 | 0.001 | 0.004 | 0.023 | 0.101 | -- | 0.001 | -- | -- | -- | 0.477 | 0.024 | -- | -- | -- | -- | -- | 0.003 | -- |
|  | 2029 | 0.001 | -- | -- | -- | -- | -- | -- | -- | -- | -- | 0.098 | -- | -- | -- | -- | -- | -- | -- |
|  | **2045** | **0.032** | -- | -- | -- | -- | -- | -- | -- | -- | -- | -- | -- | -- | -- | -- | -- | -- | -- |
|  | 2046 | 0.271 | -- | 0.003 | -- | 0.001 | -- | -- | -- | -- | -- | -- | -- | -- | -- | -- | -- | -- | -- |
|  | **2047** | **0.013** | -- | -- | -- | -- | -- | -- | -- | -- | -- | -- | -- | -- | -- | -- | -- | -- | -- |
|  | 2048 | 0.259 | -- | 0.380 | -- | 0.001 | -- | -- | -- | -- | -- | -- | -- | -- | -- | -- | -- | -- | -- |
|  | 2050 | 0.041 | -- | -- | -- | -- | -- | 0.003 | 0.153 | -- | -- | -- | -- | -- | -- | -- | -- | -- | 0.045 |
|  | **2051** | **0.011** | -- | -- | -- | -- | -- | -- | -- | -- | -- | -- | -- | -- | -- | -- | -- | -- | -- |
|  | 2052 | 0.003 | -- | 0.060 | -- | -- | -- | -- | -- | -- | -- | -- | -- | -- | -- | -- | -- | -- | -- |
|  | 2053 | 0.028 | -- | 0.047 | -- | 0.137 | 0.037 | 0.559 | 0.043 | -- | -- | -- | -- | 0.305 | -- | 0.018 | 0.486 | -- | 0.017 |
|  | 2079 | 0.001 | -- | -- | -- | -- | 0.003 | -- | -- | -- | -- | -- | -- | -- | -- | -- | -- | -- | -- |
|  | **2088** | **0.002** | -- | -- | -- | -- | -- | -- | -- | -- | -- | -- | -- | -- | -- | -- | -- | -- | -- |

Table headers correspond to the following:

GR= Golden Retriever

BRT= Black Russian Terrier

LR= Labrador Retriever

DP= Doberman Pinscher

FCT= Flat Coated Retriever

Hav= Havanese

Sam= Samoyed

GS= Giant Schnauzer

EB= English Bulldog

Bie= Biewer

IG= Italian Greyhound

AKK= Alaskan Klee Kai

SS= Shiloh Shepherd

JA= Japanese Akita

MP= Miniature Poodle

SV= Swedish Vallhound

PD= Poodle

TPD= Toy Poodle
